# Helium Suicide, a Rapid and Painless Asphyxia: Toxicological Findings

**DOI:** 10.3390/toxics10080424

**Published:** 2022-07-28

**Authors:** Anna Carfora, Raffaella Petrella, Giusy Ambrosio, Pasquale Mascolo, Bruno Liguori, Christian Juhnke, Carlo Pietro Campobasso, Thomas Keller

**Affiliations:** 1 Forensic Toxicology Unit, Department of Experimental Medicine, University of Campania “L. Vanvitelli”, Via L. Armanni, 5, 80138 Naples, Italy; raffaella.petrella@unicampania.it (R.P.); giusy.ambrosio@studenti.unicampania.it (G.A.); mascolopasquale2@gmail.com (P.M.); bruno.liguori@gmail.com (B.L.); carlopietro.campobasso@unicampania.it (C.P.C.); 2 Laboratory for Vacuum and Low Temperature Technology, University of Applied Sciences, Nibelungeplatz 1, D-60318 Frankfurt, Germany; juhnke@fb2.fra-uas.de; 3 Institute of Forensic Medicine, University of Salzburg, Ignaz-Harrer-Street, 79, A-5020 Salzburg, Austria; thomas.keller@plus.ac.at

**Keywords:** helium, asphyxia, suicide, detection and quantification

## Abstract

Suicide by helium inhalation has become increasingly common in the last few decades in Europe and the US. Inhaled-gas suicides can easily be assessed through death scene investigation and autopsy. However, helium is a colorless and odorless inert gas that unfortunately cannot be detected using standard toxicological analysis. A successful gas analysis was performed following the suicide of a 17-year-old female. For the detection of helium, central/peripheral blood samples and gaseous samples from the esophagus, stomach, and upper and lower respiratory airways (from the trachea and the primary left and right bronchia) were collected with a gastight syringe, ensuring minimal dilution. Qualitative analyses were positive in all gaseous samples. Quantitative analyses were performed using a special gas-inlet system with a vacuum by which the sample can be transferred to a mass spectrometer, reducing the risk of contamination. Helium concentrations were 20.16% from the trachea, 12.33% from the right lung, and 1.5% from the stomach. Based on the high levels of helium, the cause and manner of death were assessed as asphyxia suicide by inhalation of helium. Therefore, toxicological analyses should always be applied in order to gain evidence of inhaled gas in gaseous samples.

## 1. Introduction

In the last two decades, an increase in suicides due to gas inhalation has been observed [1,2,3,4]. Since 2000, suicide methods using a combination of plastic bag suffocation with inert gas inhalation (e.g., helium, nitrogen, nitrous oxide) have been widely reported around the world [5,6]. Helium is one of the most common inert gases involved in these events, along with propane and nitrogen [7,8,9,10,11,12]. According to Nowak et al. (2019), suicides due to helium inhalation are very common in Northern and Eastern Europe, but also in South Australia, Hong Kong, and the US. The popularity of asphyxia suicide by gas inhalation has been related to the wide spread of digital and printed publications dealing with this topic [13,14,15,16]. Similar methods are also described in literature dealing with euthanasia, self-deliverance, and assisted suicide [17,18].

In the inert gas group [19], helium is widely used to commit suicide, due to its characteristics and accessibility. It is an odorless, colorless, and nonflammable gas used to inflate balloons, which makes it extremely easy to get [20,21,22,23].

Compared to oxygen, helium has a lower density. When its air concentration increases, it replaces oxygen in the atmospheric air as well as within the lungs, causing hypoxia. With a plastic bag secured over the head by a rope, a rubber band, or adhesive tape fixed around the neck, the flow of helium into the bag can accelerate the removal of oxygen. Therefore, hypoxia is a fast process. It is estimated that loss of consciousness due to oxygen deprivation can occur in 5–10 s and within 60 s cerebral damage can be irreversible due to hypoxia [24].

Helium is very easy to breath but, in case of oxygen replacement by helium, the first symptoms of oxygen deficiency can be observed when oxygen levels go down to 12–16% from the normal oxygen concentrations of atmospheric air (21%). These symptoms are mainly represented by tachypnea, tachycardia, fatigue, and muscular coordination disorders. At lower concentrations of oxygen (6–10% approximately), loss of consciousness can occur and at levels below 6% convulsive movements and gasping breaths can anticipate the death due to brain hypoxic-ischemic injuries [7,25,26,27].

A peculiar aspect of helium inhalation is the lack of the breathing reflex or the so-called choking feeling, such that the victims do not feel the urge to breathe [15,22,28]. In fact, the breathing reflex is not triggered by oxygen deficiency, but by carbon dioxide excess, which is not present in the case of helium intoxication [28,29]. This is probably the main reason that helium is often used in euthanasia procedures [5]. Helium inhalation can cause painless asphyxia [30,31,32], which is very attractive to a potential suicide victim, as well as the availability of the gas and equipment.

Unfortunately, helium dissipates rapidly in ambient air and its presence cannot be easily detected postmortem in the blood or in tissue. In cases of helium suicide, the circumstances of disclosure of the corpse and the findings at the death scene are still of utmost importance [19,33], and examination of the cadaver can also provide very useful information [34]. However, according to various analytical processes and methods of detection, few research groups have performed toxicological analysis of helium or other inert gases on biological samples, properly collected, at autopsy. In cases of helium poisonings, the most commonly used detectors are mass spectrometers (MSs) in selected ion monitoring (SIM) mode [9,35,36,37], and thermal conductivity detectors (TCDs) [38,39,40], with the modification of mobile phase using the nitrogen or hydrogen as carrier gas.

In this case study, the cause and manner of death was assessed based on the results of the crime scene survey and autopsy findings, including the toxicological analyses. GC-MS and LS-MS/MS analyses on standard biological samples were negative for traditional drugs of abuse, pharmaceuticals, and their metabolites. A special gas-inlet system with a vacuum connected to a mass spectrometer was used for the detection and quantification of helium in gaseous samples, allowing the provision of sufficient evidence of helium inhalation. Helium-induced hypoxia was therefore assessed as the cause of death.

## 2. Materials and Methods

A 17-year-old female was found dead at home. A plastic bag over her head was fixed by a rope around the neck. Close to the body, there was a container, and a plastic tube was attached to the valve and led into the plastic bag. A forensic autopsy with toxicological analysis was requested.

### 2.1. Sampling

During the autopsy, biological samples were collected for the standard toxicological analyses. Blood, urine, bile, liver, and brain were sampled in tubes containing sodium fluoride.

Gaseous samples were also collected in order to quantify the helium concentration. For the detection of helium, central/peripheral blood samples and gaseous samples from the esophagus, stomach, and upper and lower respiratory airways (from the trachea and the primary left and right bronchia) were collected with a gastight syringe. Then, the content of the syringe was inserted in closed headspace vials in which the vacuum had already been initiated. All samples were stored in 10 mL headspace vials. These vials are the gold-standard containers for gas samples, as they are closed hermetically with an aluminum cap and a rubber seal with a magnetic crimp cap.

Intratracheal gas was sampled by gas syringe directly in the trachea after clamping. Pulmonary gases were sampled from the right primary bronchia after lung massage and bronchia clamping. Gastric gas was sampled from the stomach after esophageal and duodenal clamping, following the procedure by Varlet et al. [41]. All samples were stored at −20 °C until subsequent analysis.

### 2.2. Toxicological Analysis

A systematic toxicological analysis (STA) was performed on biological liquids, urine, and blood using gas chromatography coupled with mass spectrometry (GC-MS). In order to fill the gap with respect to thermolabile and nonvolatile analytes, analysis was also performed using liquid chromatography–tandem mass spectrometry (LC-MS/MS) for different classes of drugs of abuse, pharmaceuticals, and their metabolites. Blood was tested for alcohol and other volatile substances by headspace gas chromatography with flame ionization detection (GC-HS/FID). The method used for the analysis of biological matrices is the same reported in Carfora et al. (2018 and 2020) [42,43].

**Figure 1:**
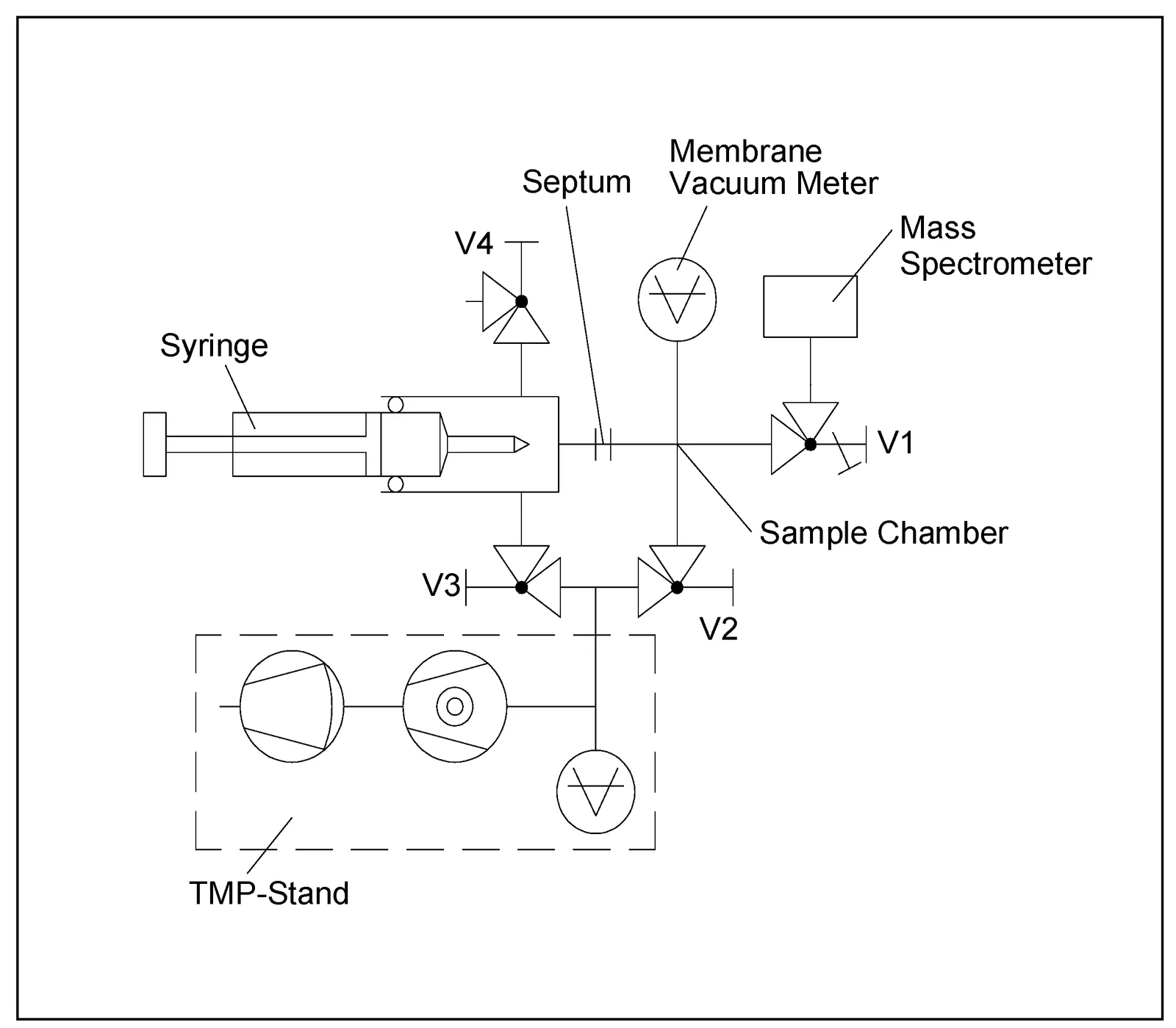
Schematic diagram of gas-inlet system.

Gas samples were analyzed at the Laboratory for Vacuum and Low Temperature Technology at the Frankfurt University of Applied Sciences, where a special gas-inlet system for the analysis of small amounts of gas is available (Figure 1). It is a receptacle for a gastight syringe that contains the gas sample to be analyzed and a vacuum system by which the sample can be transferred to a mass spectrometer with the least risk of contamination. After a proper calibration curve of helium, oxygen, nitrogen, and carbon dioxide in the spectrometer, the sample’s gas composition can be determined quantitatively with an error rate of <1%.

### 2.3. Gas-Inlet System

The syringe is pushed into a Teflon-lined guide tube until the needle protrudes into a vacuum-tight septum made of special rubber. This hermetically seals the gas sample inside the syringe. The space in front of the ultrahigh vacuum (UHV) needle valve V1 is evacuated to a pressure of <10^−1^ mbar via valve V2 with the aid of a turbo molecular pump (TMP) stand. The pressure is controlled by a gas-type independent membrane vacuum meter. Valve V2 is closed again and valve V3 is opened so that the atmospheric air contained in the space in front of the septum gets removed by the TMP stand. Only now is the septum pierced by the tip of the needle so that the gas sample can flow from the syringe into the evacuated sample chamber. The membrane vacuum meter’s display indicates if the entire gas sample is contained inside the chamber (volume ~25 cm^3^). The tip of the needle is then retracted into the septum so that any air that may enter the syringe does not flow into the chamber. In the event that the septum has a slight leak, sample gas may escape to the outside, but no atmospheric air can flow into the chamber, since the space in front of the septum is continuously being evacuated by the TMP stand. Adulteration of the gas sample’s composition is thus made impossible.

## 3. Results

On external examination of the body, asphyxia signs were observed: conjunctival petechiae and mild facial congestion, slight bruising around the mouth, skin-ligature marking all around the neck reproducing the size of the rope securing the plastic bag, and finally purplish red hypostases in the lowest anatomical areas consistent with the body position. In particular, petechiae of the conjunctiva were not extensive, but represented on both sides by a few areas of scattered pinpoint hemorrhages consistent with an increased venous pressure of the head slightly congested.

At autopsy, no relevant injuries were found, except for a diffuse congestion of internal organs along with cerebral and pulmonary edema, consistent with the suspicion of an asphyxia death. Qualitative and quantitative analyses using GC-MS and LC-MS/MS showed the following results. STA of biological fluids were negative for the most common drugs of abuse and alcohol. Qualitative analyses were positive for helium in all gaseous samples, except for the sample of the left lung, which accidentally was left open during the laboratory procedures. Quantitative analyses were positive for helium from all samples available, collected from the trachea, the right bronchia and the stomach as follows:

- Trachea: 20.16%
- Right lung: 12.33%
- Stomach: 1.5%

In all three positive samples, the helium concentration exceeded the levels normally present in air (0.0005%), but helium was not the only gas detected from the gas samples. The quantitative results of gas analysis showed also additional gases, such as oxygen, nitrogen, and carbon dioxide (Table 1).

**Figure 2:**
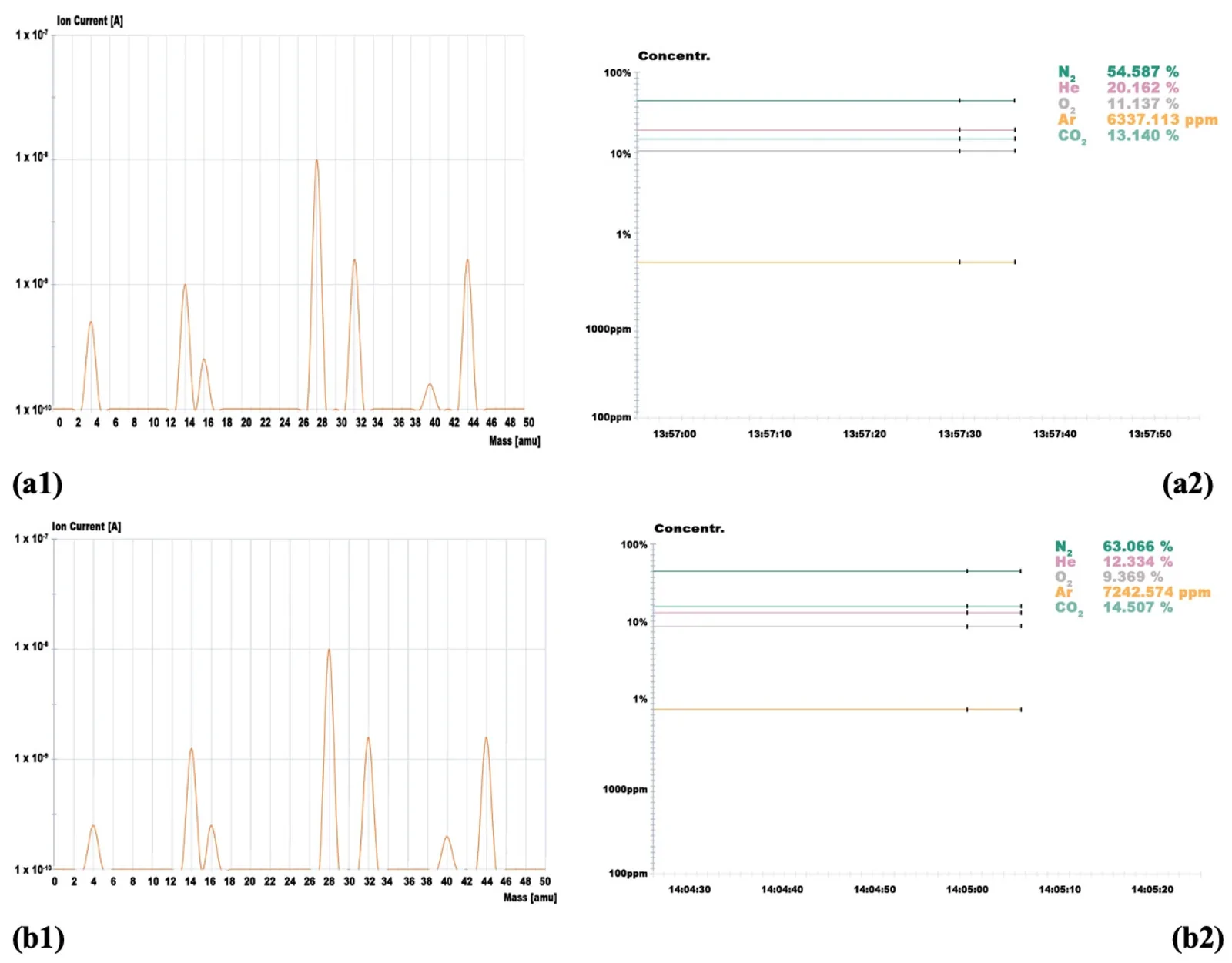
Qualitative and quantitative results of the gas analyses from the trachea sample (**a1**,**a2**) and the right lung (**b1**,**b2**).

Figure 2 shows the chromatograms of qualitative and quantitative analyses from gas samples collected from the trachea and the right lung. Based on these results, the cause of death was related to the lack of oxygen due to helium inhalation. The manner of death was suicide.

## 4. Discussion

According to Madea et al. [44], asphyxia can be identified as cause of death also in every case of exclusion of oxygen due to the “*depletion and replacement of oxygen by another gas or by chemical interference with oxygen’s uptake and utilization by the body*”. In this category, deaths by helium inhalation from a plastic bag, like in the present case, can also be included as lethal events in which oxygen is excluded and carbon dioxide, carbon monoxide, or hydrogen sulfide (toxic gases) enter the body [45,46].

The death of the young teenager has been classified as a suicidal asphyxiation by helium-induced hypoxia, based on the results of the death scene investigation and autopsy findings, including the toxicological analyses. Often, few indicators of hypoxia or suffocation can be found in victims of plastic-bag asphyxiation [47,48]. Although conjunctival petechiae and facial congestion are considered hallmarks of asphyxia deaths, they can be found in a variety of traumatic and natural deaths [49,50] as a result of increased cephalic venous pressure and hypoxic damage to endothelial cells.

The ligature mark can be the only indicator available, but it is not always present, depending on the nature of the rope or tape used to fix the plastic bag [51,52,53]. If the plastic bag and the other equipment (i.e., the rope fixing the bag over the head or the tube connecting the gas container) are removed at the death scene, the death might appear as being natural [52]. The removal of the equipment can occur when someone tries to hide the real cause of death or for financial reason, when life insurance does not cover a suicide [44,54]. Therefore, the death investigation is at risk due to inaccuracy of cause and manner of death determination. The forensic pathology community is aware that significant discrepancies between external body examination and forensic autopsy are not rare [55]. A violent death can be misclassified as natural if a death scene survey and an autopsy with toxicological analyses are not performed. An autopsy cannot be considered complete without appropriate toxicological analyses [53].

Unfortunately, the toxicological analysis of helium is not easy by standard methods, and a specific sampling procedure must be performed at the autopsy. Furthermore, a special gas-inlet system for the analysis of small amounts of gas is needed similar to the one available in the high-tech laboratory at the Frankfurt University of Applied Sciences.

Quantitative toxicological results for victims of helium inhalation are rare in the literature [39,56]. The reasons are mostly related to the heterogeneous findings due to wrong sampling at autopsy and to the fact that helium can be easily lost during storage and sample preparation. Helium might also not be detected in samples due to its loss while opening the containers to take the subsamples for toxicological analysis, as occurred in one of our samples (the sample collected from the left bronchus).

When the sampling method is carried out properly, helium can still be detected even 3 days after death [39]. In a case reported by Auwaerter et al. [56], helium was detected in samples not only from lung tissue but also from the brain and heart blood in such high concentrations that exceeded those normally present in air (0.0005%) by up to four orders of magnitude. Our results also demonstrate that the helium concentration detected was higher than those reported in literature.

## 5. Conclusions

Asphyxia by helium inhalation may not leave any physical signs useful to assess the cause and manner of death. Sophisticated forensic toxicological analyses will be able to verify helium exposure prior to death. In suspected unnatural deaths, especially those related to euthanasia or assisted suicide, an accurate sampling procedure using a gastight syringe and closed headspace vials must be considered at autopsy. Helium can be lost if sampling is not performed and processed properly at the laboratory. Furthermore, the toxicological analysis of helium is not easy to perform by standard methods. A special gas-inlet system should be considered crucial to receive reliable results.

## Figures and Tables

**Table 1:** Results of qualitative and quantitative gases analyses.

	Trachea	Right Bronchus	Stomach
Qualitative Analysis	Positive	Positive	Positive
Helium (He)	20.16%	12.33%	1.5%
Oxygen (O_2_)	11.14%	9.37%	—
Nitrogen (N_2_)	54.59%	63.06%	—
Carbon Dioxide (CO_2_)	13.14%	14.50%	—
