# Correction: Carfora et al. Helium Suicide, a Rapid and Painless Asphyxia: Toxicological Findings. *Toxics* 2022, *10*, 424

**DOI:** 10.3390/toxics13070591

**Published:** 2025-07-15

**Authors:** Anna Carfora, Raffaella Petrella, Giusy Ambrosio, Pasquale Mascolo, Bruno Liguori, Christian Juhnke, Carlo Pietro Campobasso, Thomas Keller

**Affiliations:** 1 Forensic Toxicology Unit, Department of Experimental Medicine, University of Campania “L. Vanvitelli”, Via L. Armanni, 5, 80138 Naples, Italy; raffaella.petrella@unicampania.it (R.P.); giusy.ambrosio@studenti.unicampania.it (G.A.); mascolopasquale2@gmail.com (P.M.); bruno.liguori@gmail.com (B.L.); carlopietro.campobasso@unicampania.it (C.P.C.); 2 Laboratory for Vacuum and Low Temperature Technology, University of Applied Sciences, Nibelungeplatz 1, D-60318 Frankfurt, Germany; juhnke@fb2.fra-uas.de; 3 Institute of Forensic Medicine, University of Salzburg, Ignaz-Harrer-Street, 79, A-5020 Salzburg, Austria; thomas.keller@plus.ac.at

**Text Correction**

The authors have made minor revisions to the wording in the published manuscript [1] in response to concerns raised by readers regarding the sensitivity of the topic discussed. Corrections have been made to the Abstract and Introduction.

*Deleted Sentence*

In Section 1 Introduction: In these references, the readers can find all the instructions useful, already applied by the victims who reported the methods on their laptops and smartphones.

*Revised Sentences*

In Abstract: Suicide by helium inhalation has become increasingly common in the last few decades in Europe and the US.

In Section 1 Introduction: Similar methods are also described in literature dealing with euthanasia, self-deliverance, and assisted suicide [17,18].

The authors state that the scientific conclusions are unaffected. This correction was approved by the Academic Editor. The original publication has also been updated.
